# Modified Dose of Radioactive Iodine Therapy in a Patient With Thyroid Cancer on Peritoneal Dialysis: Case Report and Review of Literature

**DOI:** 10.7759/cureus.44754

**Published:** 2023-09-05

**Authors:** Ahmad Makeen, Rawan Alzahrani, Sarah Dahlan, Raad Alwithenani

**Affiliations:** 1 Nephrology, King Abdulaziz Medical City, Ministry of National Guard Health Affairs, Jeddah, SAU; 2 Endocrinology and Diabetes, King Abdulaziz Medical City, Ministry of National Guard Health Affairs, Jeddah, SAU; 3 College of Medicine, King Said Bin Abdulaziz University for Health Sciences, King Abdullah International Medical Research Center, Jeddah, SAU

**Keywords:** kidney transplant, end-stage renal disease (esrd), radioactive iodine activity, peritoneal dialysis (pd), thyroid cancer

## Abstract

Thyroid malignancy is common among patients with renal impairment compared with the general population. Treatment involves surgical resection and radioactive iodine therapy (RAI) in high-risk patients. As a result of impaired iodine clearance in those with no residual kidney function, the determination of appropriate iodine dose is challenging. Evidence is lacking, and all previous reports are based on case studies with no universally accepted protocol.

We describe the case of a 30-year-old woman with end-stage renal disease on peritoneal dialysis (PD) who was diagnosed with papillary thyroid cancer while undergoing a pre-kidney transplant workup. She had a total thyroidectomy with modified radical neck dissection followed by a reduced-dose radioactive iodine therapy of 30 mCi based on her residual kidney function. Her PD prescription was adjusted to achieve a 2 L ultrafiltration daily. One year follow-up confirmed no evidence of residual nor recurrent disease.

High-risk patients with differentiated thyroid malignancy require adjuvant radioactive iodine therapy. The optimal dose of RAI in the end-stage renal disease population is controversial. There are no clear guidelines available for patients with end-stage kidney disease including patients on peritoneal dialysis. Reduced dose therapy is probably effective in achieving the goals of therapy, with lower toxic risk to internal organs. Determining the appropriate schedule of each dialysis session in relation to RAI, the specific replacement prescription, and establishing a safe environment for medical staff dealing with such patients is important to consider. This article aims to highlight the need to establish a standardized protocol among patients with reduced kidney function treated with iodine therapy.

## Introduction

Thyroid cancer is the most frequent endocrine malignancy worldwide. Its prevalence has increased over the last few decades, which is mainly attributed to improvements in diagnostic accuracy; however, its mortality rate remains stable [1,2]. Epidemiological studies have shown that thyroid cancer is more predominant in women [3]. The management of thyroid tumors involves total or partial thyroidectomy followed in some patients by radioactive iodine therapy (RAI). RAI is used in most differentiated thyroid cancers to facilitate the elimination of residual thyroid tissue and to target possible locoregional or distant metastasis [4]. 

Chronic kidney disease (CKD) is a major health concern worldwide. Around 15% of the United States adults are estimated to have CKD [5]. Its prevalence among Saudi adults is reported to be approximately 13.8% [6]. The incidence of thyroid cancer is higher in patients with CKD than in the general population [7,8]. The treatment of thyroid cancer in patients with severe renal impairment and end-stage renal disease (ESRD) is challenging.

Most radioactive iodine is excreted by the kidneys; therefore, patients with ESRD are expected to retain a higher level. This is linearly associated with increased toxicities, mainly in the bone marrow [9]. Compared with healthy individuals, where iodine clearance is reported to be more than 50% after 24 h of the RAI, the clearance was only 11% in patients with ESRD within the same period [10]. Higher concentrations of iodine were observed in more than 80% and 90% of the patients undergoing hemodialysis (HD) and peritoneal dialysis (PD), respectively [11]. As a result, different protocols, including a reduction of approximately 30% in the dose of RAI, were adopted and used in ESRD patients by a few centers to avoid toxicity [12,13].

The appropriate RAI dose and dialysis timing are controversial in dialysis patients with thyroid cancer compared to those with normal kidney function. To date, no universal recommendations have been made, and evidence is still lacking. While data among HD patients are rare, reports regarding patients on PD are scarce.

Here, we present the case of an adult woman with papillary thyroid cancer who underwent continuous ambulatory PD (CAPD) and was treated with total thyroidectomy followed by RAI 131I therapy. To the best of our knowledge, this is the first reported case in Saudi Arabia.

## Case presentation

We report the case of a 30-year-old woman with ESRD undergoing CAPD who was referred to our tertiary hospital for further evaluation and management of underlying papillary thyroid cancer (PTC). The cause of renal failure was unknown, although she had been regularly seeing a nephrologist for renal impairment since childhood. A thyroid nodule was identified on neck ultrasound (US) as part of her pretransplant workup at her primary hospital. The patient had no neck compressive symptoms like dysphagia or shortness of breath. She was euthyroid clinically. The patient had no family history of thyroid cancer or radiation exposure. She was a nonsmoker, unemployed, and living with her parents. Her weight is 52 Kg with a body mass index (BMI) of 30. 

She had been undergoing CAPD for 10 years, and her regimen included a total of three exchanges (two exchanges of 2.3% and one exchange of 1.5%) in addition to icodextrin. The patient had no urine output. The usual ultrafiltration rate was approximately 700 mL/day. 

Fine-needle aspiration (FNA) of the thyroid nodules at her primary hospital revealed features of PTC (Bethesda V). She underwent total thyroidectomy with modified radical neck dissection 6 months before consulting our hospital. Histopathology confirmed the diagnosis of classic multifocal papillary thyroid carcinoma with a tumor size of 1.5 × 1 × 0.6 cm with no lymphovascular invasion or extrathyroidal extension. Two of the seven lymph nodes were positive for metastatic carcinoma with the largest deposition of 0.8 cm. Her pathological Tumor-Node-Metastasis (pTNM) pathological stage was pT1b N1a Mx. 

Her final thyroglobulin (TG) level before referral was 0.279 ng/ml, with negative TG antibodies and a serum thyroid stimulating hormone (TSH) level of 1.2 mIU/L (0.6-5.8 mIU/L). Post-operative thyroid US revealed no evidence of residual thyroid tissue or recurrence. Based on the overall presentation and pathological findings, our patient was stratified based on the American Joint Committee on Cancer as stage 1 PTC with an intermediate risk of persistence/recurrence based on the American Thyroid Association (ATA) criteria.

A tumor board meeting was carried out, where it was agreed upon to proceed with adjuvant RAI 131I therapy to target potential microscopic residual disease particularly as she was planned to undergo a kidney transplant. A multidisciplinary team, including an endocrinologist, nephrologist, and PD nurse, was arranged. A nuclear medicine team and radiation officers were also on board. The patient was prepared with a low iodine diet starting 2 weeks before RAI. She received two doses of recombinant human thyrotropin 48 hours before therapy as per protocol. No standardized radiation dose has been recommended in the literature for patients with PD, although a few case reports have suggested a reduced dose with good results. In patients with normal kidney function and intermediate ATA risk for recurrence, the appropriate dose ranges between 75-150 mCi. Our patient received an empirical reduced dose of 30 mCi, given the fact of her renal impairment, lack of urine output, and risk of internal organ toxicity, particularly to the bone marrow. During admission, her PD prescription was adjusted to increase her ultrafiltrate as she has no urine output. The exchanges were increased to 2.3%, which helped achieve a 2 L ultrafiltration daily for the next 7 days which is approximate to the normal urine output of a person with normal kidney function. This prescription aimed to minimize possible risks associated with higher iodine levels. The patient was able to perform her usual sessions by herself. All equipment used including PD solutions were carefully eliminated and expelled by expert medical staff to minimize the risk of radiation exposure. 

**Figure 1:**
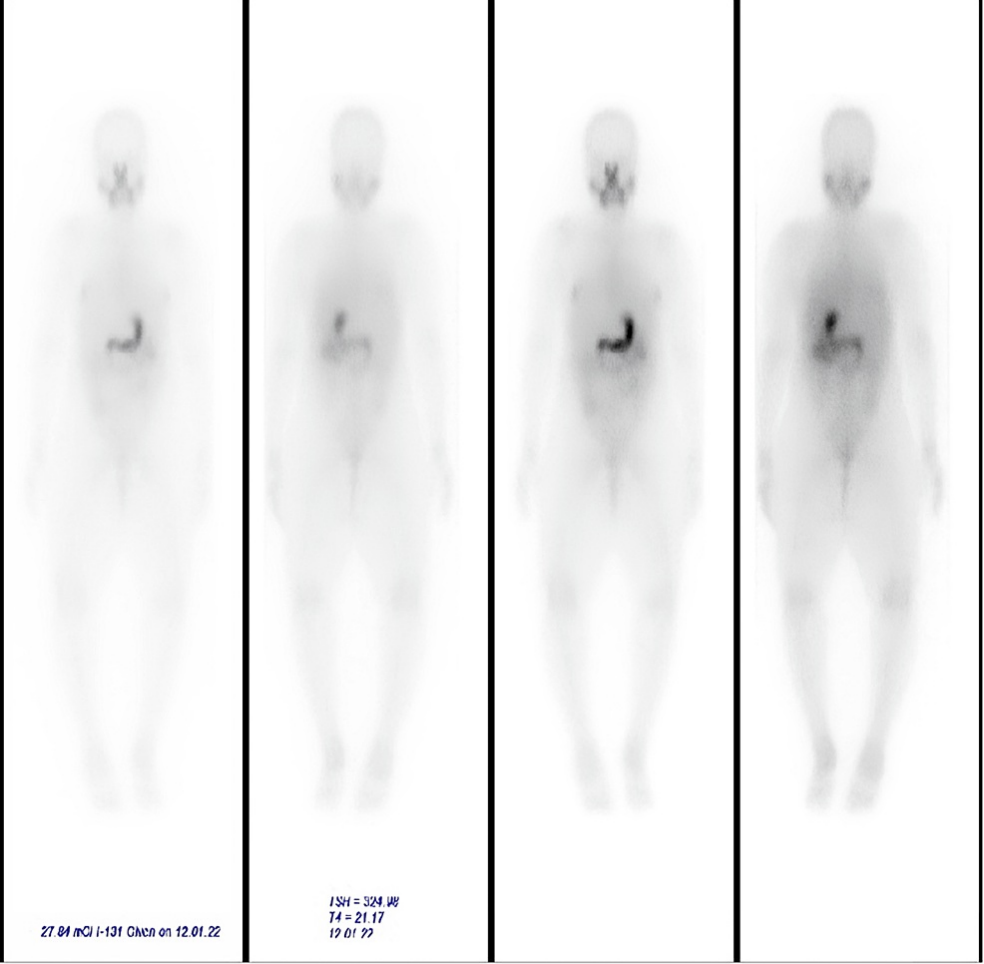
131I WBS with no evidence of residual disease or metastasis. WBS: whole-body scan

The day after RAI, the patient underwent a** **131I whole-body scan (WBS) looking for any evidence of residual or persistent disease, which revealed the absence of thyroid bed residual tissues; no iodine-131 avid regional or distant metastasis; and physiological activity in the salivary glands, stomach, and colon (Figure 1). Her stimulated thyroglobulin was 0.5 ng/ml.

She was discharged from the hospital shortly after the therapy when she reached a safe iodine threshold, as advised by the nuclear medicine team. The patient underwent frequent surveillance with the measurement of TG levels and antibodies (Table 1). 

**Table 1:** Sample of the patient’s blood laboratory results RAI: radioactive iodine therapy; TSH: thyroid-stimulating hormone

		12-months after RAI dose	One-month after RAI dose	Before Receiving RAI		
Test	Reference value	1/2023	2/2022	1/2022	11/2021	7/2021
Thyroglobulin Antibody	5-100 IU/mL	25.01	25.18	26.88	27.03	7.4
Thyroglobulin	0.2-70 ng/mL	0.14	0.1	0.5	-	0.279
TSH	0.6 ~ 5.8 mIU/L	0.2	0.10	268.73 (Effect of thyrotropin)	-	1.26

In the following year, her blood counts indicated no evidence of bone marrow toxicity which has been regularly checked every 3 months. She continued levothyroxine targeting a TSH level between 0.1-0.5 as part of the suppressive therapy protocol. 

## Discussion

The association between renal impairment and thyroid disorders is well established. This relationship involves several hormonal changes, including a decrease in total and free T3 levels due to decreased conversion of T4 to T3 [14]. Serum iodine levels, multinodular goiter, and thyroid cancer were significantly increased in patients with advanced CKD [15,16].

RAI therapy is an important aspect of the management of most differentiated thyroid cancers. The decision to proceed with such therapy depends on ATA risk groups and the results of the thyroglobulin level and neck ultrasound obtained after surgery. The ATA guidelines recommend iodine ablation for patients with intermediate and/or high ATA-risk thyroid cancer [4]. The aim of RAI includes ablation of remnant thyroid tissues, adjuvant treatment in case of sub-clinical micrometastatic disease, or treatment of metastatic and apparent residual thyroid cancer. Higher doses are required in the cases of adjuvant or metastatic disease, especially with normal kidney functions. 

Iodine ablation is challenging in patients with ESRD. Following the administration of 131I, most of the blood iodine (approximately 75%) is excreted and cleared by the kidneys [11]. A reduction in the estimated glomerular filtration rate leads to an increase in the serum and total amount of iodine in the body, which exceeds the acceptable dose to the bone marrow and causes hematopoietic toxicities. An increase in the half-life of iodine in the body increases the risk of irradiation to family members and healthcare providers. The main obstacles faced by physicians are determining the appropriate dose for these patients, the interval between dialysis sessions after receiving the dose, and whether a repeated dose is necessary. Most current practices depend on case studies or case series, with no established universal guidelines. While a few studies have reported a suitable approach for dealing with patients on HD, data on patients receiving PD are limited. The appropriate dose of RAI used to treat thyroid cancer in ESRD is based on individual dosimetry studies or empirical dose regimens. The patient we presented received an empirical reduced dose of 131I based on an extensive review of previously published similar cases. No evidence of disease recurrence nor any notable toxicities over the short-term follow-up occurred. 

Among patients on HD, Mello and colleagues recommended administering 25% of the total calculated dose 48 hours after the last dialysis session [17]. Similarly, the treatment of six hemodialysis patients with thyroid cancer using a 30% lower than the normal RAI dose achieved adequate therapeutic efficacy while balancing the risk of bone marrow toxicity [13]. In contrast, other reports have recommended full-dose therapy, especially in patients with advanced metastatic disease, or repeating the normal dose if a single dose is proven to be effective and safe [18,19]. The timing of HD after the dose also varied between studies, which included daily dialysis for 5 days or 48 hours after the RAI dose. In general, all suggested protocols demonstrated efficacy and safety for both patients and staff, but still with no universal agreement.

Management protocols involving ESRD patients undergoing PD are even more limited. All evidence in the literature is derived from case reports. Most patients received a reduced dose compared to those with normal kidney function, regardless of the approach applied (empirical/dosimetry-based). The first study evaluated radioiodine kinetics in two CAPD patients with thyroid cancer estimated five times longer serum iodine half-life [20]. In that study and based on dosimetry calculations, both CAPD patients received five times RAI dose reduction with 29.9 mCi and 26.6 mCi, which is found to be equivalent to 150 mCi in patients with normal kidney function. Similarly, Toubert et al. used a lower RAI dose of approximately 22 mCi, which was found to be safe and effective [21]. However, higher doses have been reported. Alevizaki et al. managed a patient on intermittent PD with differentiated thyroid cancer in which the patient received a total dose of 40 mCi and was recurrence-free over the next 2 years [22]. Willegaignon et al. used a larger dose of 100 mCi; however, this dose was calculated using a specific dosimetry of that patient [23] (Table 2). 

**Table 2:** Previous studies involving patients with differentiated thyroid cancer who underwent peritoneal dialysis. PTC: Papillary Thyroid Cancer, FTC: Follicular Thyroid Cancer, CAPD: Continuous Ambulatory Peritoneal Dialysis, IPD: Intermittent Peritoneal Dialysis.

Author	Number of patients	Histopathology	RAI dose	PD mode	Dialysis dose	Reported toxicity
Kaptein et al. [20] (2000)	2	PTC	29.9/26.6 mCi	CAPD	3-5 exchanges of 2 L/day	None
Toubert et al. [21 (2001)	1	FTC	22 mCi	CAPD	4 exchanges of 2L/day	None
Alevizaki et al. [22] (2006)	1	PTC	40 mCi	IPD	Not mentioned	None
Willegaignon et al. [23] (2010)	1	PTC	100 mCi	CAPD	Once Daily (no details)	Not mentioned

An important aspect of managing ESRD with thyroid cancer that requires RAI therapy is to maintain the safety of healthcare providers. While this might not represent a concern for patients on PD because patients are usually able to perform their regular dialysis sessions independently, patients on HD represent a major concern. The average exposure per each session of HD is ranging between 3-4 hours. Medical staff should wear protective clothing and work behind a shield. Modarresifar and colleagues adopted a safety protocol for patients on HD in which each session should be carried out by three teams. The first team should be responsible for preparing and initiating the procedure. The second and third teams will perform the procedure and prepare the equipment (including sterilization) for subsequent sessions [24]. To avoid toxic radiation effects on family members, the average dose rate at a distance of 1 m at the time of discharge should be minimal. This was reported to be between 15-25 uSV/hour in a few studies [13].

Our patient presented with differentiated thyroid cancer (DTC) and metastatic lymph node invasion. Based on her risk stratification, and as the aim was to eliminate any probable microscopic residual disease particularly as she is planned for a future kidney transplant, the decision of the multidisciplinary team was to proceed with adjuvant RAI therapy. The fact that the patient had no urine output highlights the importance of choosing the appropriate dose to prevent possible toxicities. She received 30 mCi RAI empirically based on a review of previous studies. This is different than what is usually given to patients with the same risk stratification and normal kidney function in which a higher dose of up to 150 mCi is required. Her usual dialysis prescription was adjusted to use a PD solution concentration of 2.5% and five exchanges to increase her ultrafiltration up to 2 L daily which is approximate to the 24-hour adequate urine output for an adult with normal renal function. WBC scan confirmed no residual disease. A 1-year follow-up including clinical examination, close follow-up of the thyroglobulin level, and complete blood counts confirmed a complete response with no evidence of recurrence or harmful effects.

Based on our reported case, along with the evidence from previous case reports, we believe that a standardized reduced RAI dose in patients with ESRD and DTC including those on regular PD is safe and effective and can prevent future recurrence by eliminating any residual or metastatic disease. 

## Conclusions

The determination of the appropriate RAI dose for thyroid cancer and renal impairment is controversial. Patients likely require a dose reduction because iodine clearance is impaired in such patients. When possible, the dosimetry approach appears to be the most suitable path to ascertain the proper management plan. At present, all evidence has been derived from case reports/series with small numbers of patients. The development of a standardized protocol for patients with ESRD and thyroid cancer is essential. Similarly, an important aspect in managing such patients is establishing a clear pathway which guarantee the maximum safety precautions for the medical staff and family members. 
